# Modeling congenital cytomegalovirus infection in human inner ear organoids

**DOI:** 10.1016/j.isci.2026.118148

**Published:** 2026-11-06

**Authors:** Lucia C.M. Grijpink, Wouter H. van der Valk, John C.M.J. de Groot, Heiko Locher, Ann C.T.M. Vossen

**Affiliations:** 1 Leiden University Center for Infectious Diseases (LUCID), Medical Microbiology and Infection Control, Leiden University Medical Center, Leiden, the Netherlands; 2 Department of Otorhinolaryngology and Head & Neck Surgery, Leiden University Medical Center, Leiden, the Netherlands; 3 The Novo Nordisk Foundation Center for Stem Cell Medicine (reNEW), Leiden University Medical Center, Leiden, the Netherlands

**Keywords:** cytomegalovirus, organoid model, inner ear, congenital infection, hearing, PDGFRA, EGFR, NRP2

## Abstract

Congenital infection with human cytomegalovirus is a leading cause of congenital hearing loss. Investigating its effects is challenging due to the virus’s strict species specificity, limiting the relevance of animal models. This study aimed to establish a human-specific model of cytomegalovirus infection in the developing inner ear using inner ear organoids derived from pluripotent stem cells. Organoids were cultured for 75 days, vibratome sectioned into 300-μm slices, and exposed to laboratory or clinical strains of human cytomegalovirus for 7 or 21 days. Viral replication was confirmed, with successful infection by clinical strains requiring a minimum viral titer. Immunofluorescence revealed targeted infection of nonsensory epithelial cells and mesenchymal cells, with occasional infection of hair cells and neurons. This model replicates the key aspects of congenital cytomegalovirus infection, providing a scalable human platform to study viral pathogenesis, identify susceptible cell types, and explore targeted therapies.

## Highlights

• Human inner ear organoids can provide a model for studying congenital CMV infection
• The full CMV replication cycle occurs within the organoid model
• Otic cells within organoids express cytomegalovirus host receptors
• Clinical and laboratory CMV strains predominantly infect non-sensory epithelial cells

## Graphical abstract

**Figure:**
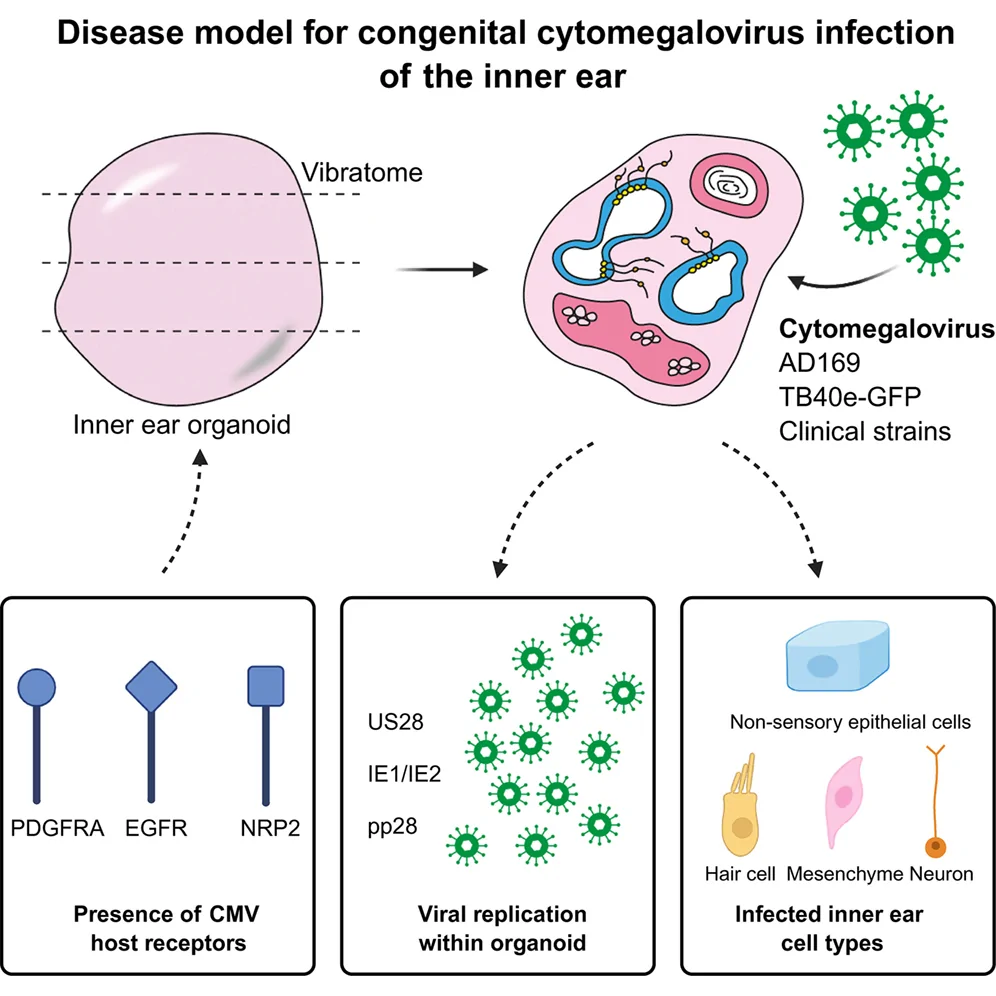

## Teaser

Neuroscience; Virology; Stem cells research

## Introduction

Human cytomegalovirus (hCMV) is the most common cause of congenital infections worldwide, with a birth prevalence ranging from 0.5% in high-income countries to up to 6% in low-income countries.^1^^,^^2^ This congenital infection is the leading cause of nongenetic sensorineural hearing loss, affecting 12.6% of infected children.^3^ Additionally, vestibular dysfunction is frequently observed and may occur at an even higher rate.^3^^,^^4^^,^^5^ Despite its profound disease burden, the underlying mechanisms of hCMV-induced inner ear damage remain poorly characterized, limiting the advancement of effective preventive and therapeutic strategies.

Animal models such as mice, guinea pigs, and rhesus macaques have provided valuable insights into CMV pathogenesis.^6^^,^^7^^,^^8^ These studies have shown that infection can increase blood-labyrinth barrier permeability, trigger immune cell infiltration, and lead to the loss of spiral ganglion neurons.^9^^,^^10^^,^^11^^,^^12^^,^^13^^,^^14^^,^^15^ However, because CMV is highly species specific, hCMV does not replicate in nonhuman hosts.^16^^,^^17^ Furthermore, murine models cannot recapitulate transplacental transmission, a key hallmark of congenital infection.^6^^,^^18^ Anatomical and embryological differences introduce additional barriers. As a result, findings from animal studies do not fully translate to human infection.

Human studies face equally significant challenges. Fetal inner ear tissue is rarely available, and the organ’s limited regenerative capacity constrains the development of *ex vivo* models. Most postmortem analyses capture late-stage infection and show that hCMV primarily targets nonsensory epithelial cells in the cochlea (such as the stria vascularis and Reissner’s membrane) and vestibular system (such as dark cells).^19^^,^^20^^,^^21^^,^^22^^,^^23^^,^^24^ Less frequently, hCMV is detected in the sensory epithelium, including hair cells and neurons.^19^^,^^23^^,^^24^ However, the specific cell types infected early in disease, and the sequence of viral spread within the inner ear, remain unknown. Therefore, there is a need for human-based model systems that capture the cellular diversity and architecture of the developing inner ear.

Organoids derived from human induced pluripotent stem cells (hiPSCs) address this need. Brain organoids have already become an important tool for studying the effects of congenital CMV infection.^25^^,^^26^^,^^27^^,^^28^^,^^29^^,^^30^ Recently, inner ear organoids (IEOs) have been developed, which recapitulate the first-trimester inner ear, when infection carries the highest risk of causing inner ear symptoms.^31^^,^^32^^,^^33^ These organoids contain otic vesicles, comprising vestibular sensory epithelium, neurons that form synapses with hair cells, and diverse otic nonsensory and mesenchymal cell types.^34^^,^^35^ These vesicles are situated within a dense, multilineage cell aggregate dominated by mesenchyme and chondrocytes. Although IEOs have been used to model other viral infections,^36^ their potential for studying hCMV pathogenesis remains unexplored.

Here, we establish a model of hCMV infection in IEOs using both well-characterized laboratory strains (AD169 and TB40e-GFP) and clinical isolates. The use of clinical isolates is particularly important, as they retain the tropism of circulating strains and have not undergone the extensive passaging and potential genetic adaptation that can alter viral behavior.^37^ This approach recreates key pathological features of congenital infection, providing a platform to investigate the molecular and cellular mechanisms of hCMV-induced inner ear damage and to support the development of targeted interventions.

## Results

### HCMV strains replicate within the IEOs

To assess whether hCMV can replicate in IEOs, we inoculated vibratomed sections of IEOs that had been in culture for 75 days. At this time point, these IEOs resemble the late first trimester, a time point in which congenital CMV infection can be very pathogenic. After inoculation, we cultured them for another 7 or 21 days for the infection to spread (Figure 1A). Supernatant was collected immediately before each medium change, and viral DNA was quantified by qPCR (Figure 1B). Viral genomes were abundant on day 1 because the inoculum had been added the preceding day; by day 4, the load fell sharply after unbound virions were removed during the first medium change. From that point onward, CMV DNA levels produced by the replication-competent strains (TB40e-GFP and AD169) rose steadily and diverged from those of the heat-inactivated control. Generalized estimating equation modeling confirmed that both strains sustained significantly higher viral loads than the control throughout the experiment (strain × time interaction, *p* < 0.001), with the gap reaching ∼4.5–5 log10 IU ml^−1^ on day 19 (Figure 1B).

**Figure 1:**
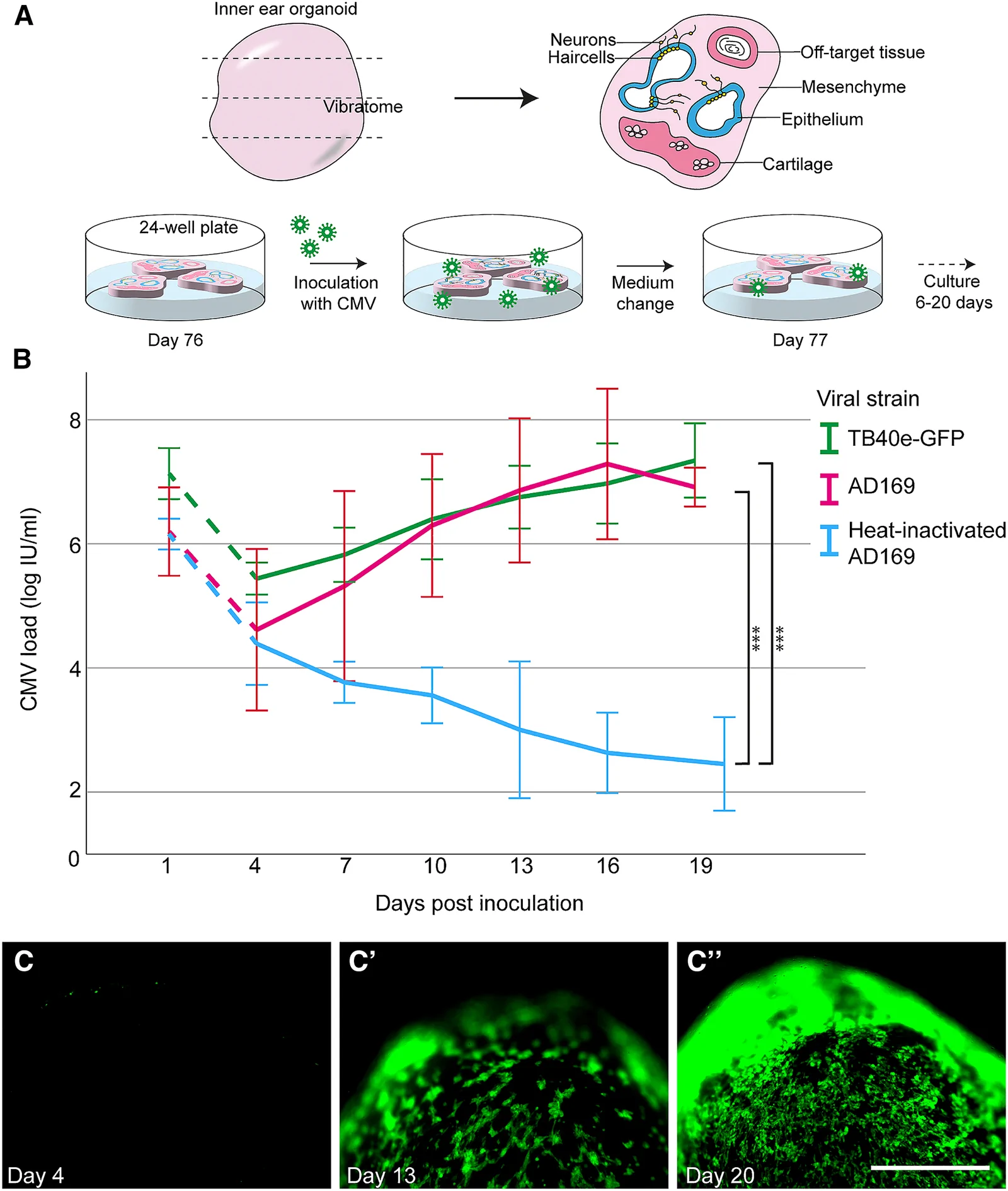
Viral load and GFP signal increase reveal CMV replication in inner ear organoids (A) Graphic depiction of the inoculation protocol. Inner ear organoids (IEO) contain otic vesicles, comprising vestibular sensory epithelium, neurons that form synapses with hair cells, and diverse otic nonsensory and mesenchymal cell types. These vesicles are situated within a dense, multilineage cell aggregate dominated by mesenchyme and chondrocytes as well as off-target tissue such as skin and muscle. IEOs were sectioned using a vibratome to expose internal structures. Following sectioning, IEO slices were inoculated with CMV. After a subsequent media change, the cultures were maintained for up to 21 days. (B) Mean CMV viral load of AD169, TB40e-GFP, and heat-inactivated AD169 over time, measured by qPCR. Supernatant samples were collected prior to each medium change. IEOs were inoculated on day 0, with the first sampling and medium change occurring on day 1. This initial time point includes the inoculum to provide a baseline for total viral load. The dashed line connecting the first and second time points indicates the transition from the inoculation phase to the replication phase. This drop reflects the clearance of non-internalized particles rather than a decrease in replication efficiency. No additional virus was added thereafter. Representative graph of 39 wells during 2 experiments. ⁎⁎⁎*p* < 0.001. Error bars: ± 2SD. (C) Representative images showing GFP signal in an IEO inoculated with TB40e-GFP at days 4, 13, and 20. Scale bar, 1,000 μm.

Replication could further be tracked using the GFP signal from TB40e-GFP-infected aggregates throughout the culture period (Figure 1C). GFP intensity increased over time, indicating progressive viral spread within the aggregate over the 21-day post-inoculation period. Together, this shows that the IEOs are permissive to CMV infection and that CMV can replicate in this culture model.

### Inoculation of vibratomed IEO slices facilitates viral infection of inner ear cell types

To verify whether the otic vesicles within the IEO had been exposed to the inoculum, aggregates were sectioned at 7 or 21 days post-inoculation and analyzed by immunohistochemistry (IHC). At day 7, AD169-inoculated IEOs showed infection localized primarily to cells at the aggregate periphery (Figure 2A). By day 21, infected cells were detected deeper within the IEO core (Figure 2B). The percentage of infected peripheral cells also increased significantly between days 7 and 21 in both TB40e-GFP- and AD169-inoculated IEOs (Figure 2C). No infection was observed in aggregates exposed to heat-inactivated AD169 (Figure 2D). When the protocol was applied to intact, nonvibratomed organoids, the virus was unable to penetrate the organoid core within 20 days (Figure S1). Importantly, vibratome sectioning provided access to the internal otic structures without disrupting key aspects of the 3D tissue architecture, as evidenced by the presence of distinct SOX10^+^ epithelial vesicles containing MYO7A^+^ hair cells (Figures 2A, 2B, and 2D).

**Figure 2:**
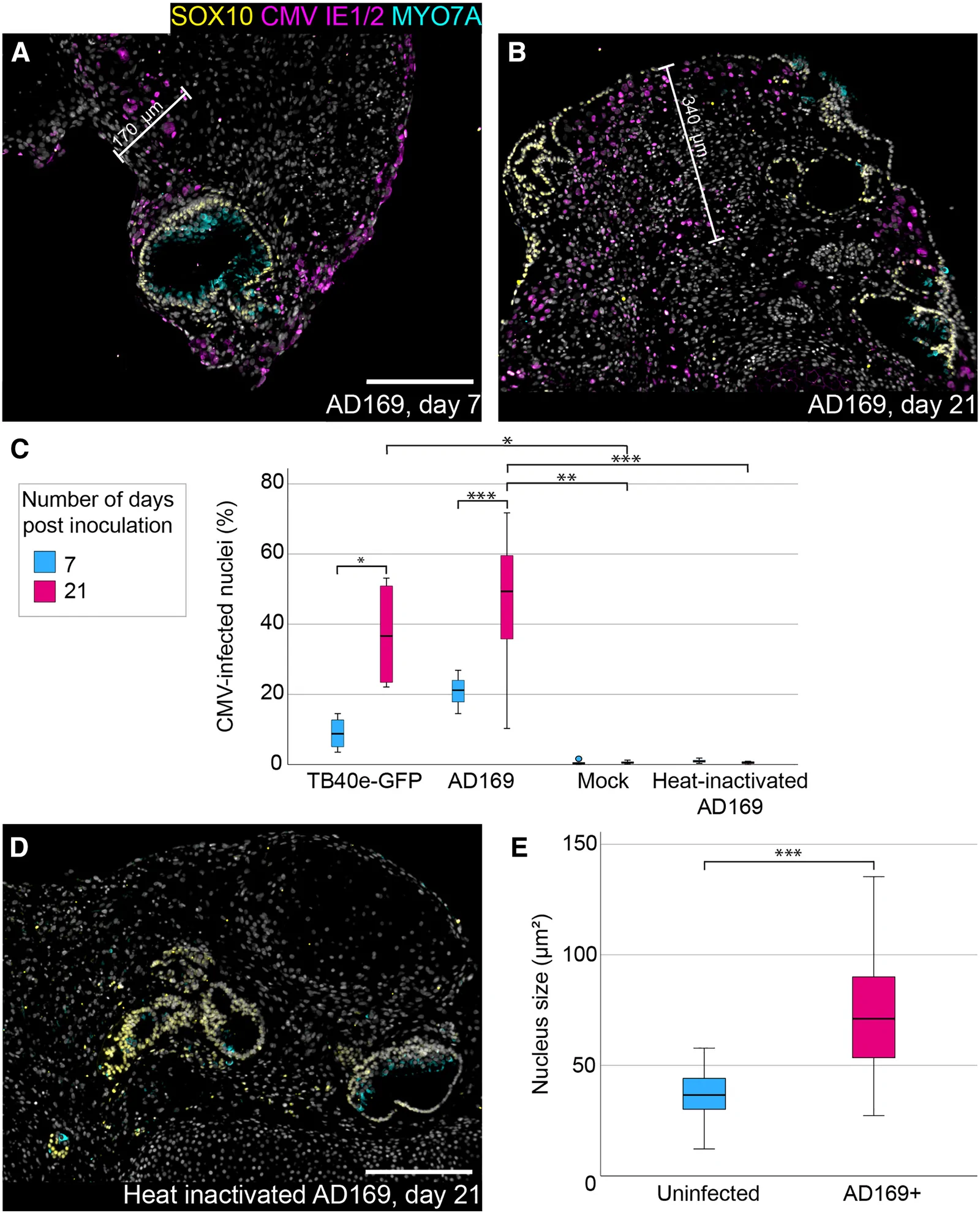
hCMV progressively spreads within IEOs and causes nuclear enlargement (A and B) IHC of IEO slices inoculated with AD169 for (A) 7 or (B) 21 days. Otic vesicles contain epithelial cells (SOX10^+^, yellow) and hair cells MYO7A^+^ (cyan). The measurement bar indicates the depth of CMV IE1/IE2 penetration (magenta) within the IEO core. Scale bar, 200 μm. (C) Boxplot showing the percentage of infected cells following exposure to AD169, TB40e-GFP, heat-inactivated AD169, or medium only (mock). Infection was quantified based on CMV IE1/IE2 protein expression relative to total nuclei (DAPI staining) in immunohistochemistry images. At least three images per condition were analyzed. Statistical significance was assessed using 2-way ANOVA, confirmed with Mann-Whitney U when normality was not met. ⁎*p* < 0.05, ⁎⁎*p* < 0.01, ⁎⁎⁎*p* < 0.001. (D) IHC of IEO slices inoculated with heat-inactivated AD169. Otic vesicles contain epithelial cells (SOX10^+^, yellow) and hair cells (MYO7A^+^, cyan). CMV IE1/IE2 (magenta) protein was not detectable. Scale bar, 200 μm. (E) Boxplot comparing nuclear size of AD169-infected cells to uninfected cells within the same otic vesicle in μm^2^. Infected cells exhibited a significant increase in nuclear size (⁎⁎⁎*p* < 0.001, Mann-Whitney U). The representative image used for this analysis is shown in Figure S2.

In summary, vibratomed IEOs cultured for 21 days achieved effective viral exposure of otic vesicles.

A pathognomonic sign of CMV infection is nuclear enlargement. To assess this in our model, we measured the sizes of CMV IE1/IE2^+^ nuclei within otic vesicles and compared them to adjacent CMV IE1/IE2^−^ nuclei from the same vesicles (Figures 2E and S2). Infected nuclei were significantly larger than neighboring noninfected nuclei.

### Infected inner ear cell types support the full replication cycle of hCMV

To determine whether hCMV was replicating within the otic vesicles, we assessed the expression of viral proteins associated with different stages of the replication cycle using the Quantitative Temporal Viromics (QTV) classification.^38^ IEOs inoculated with AD169 and TB40e-GFP and cultured for 21 days show expression of immediate-early protein CMV IE1/IE2 (Tp1), early protein US28 (Tp4), and late protein pp28 (Tp5) (Figure 3). This indicated progression through the different stages of the replication cycle of hCMV.

**Figure 3:**
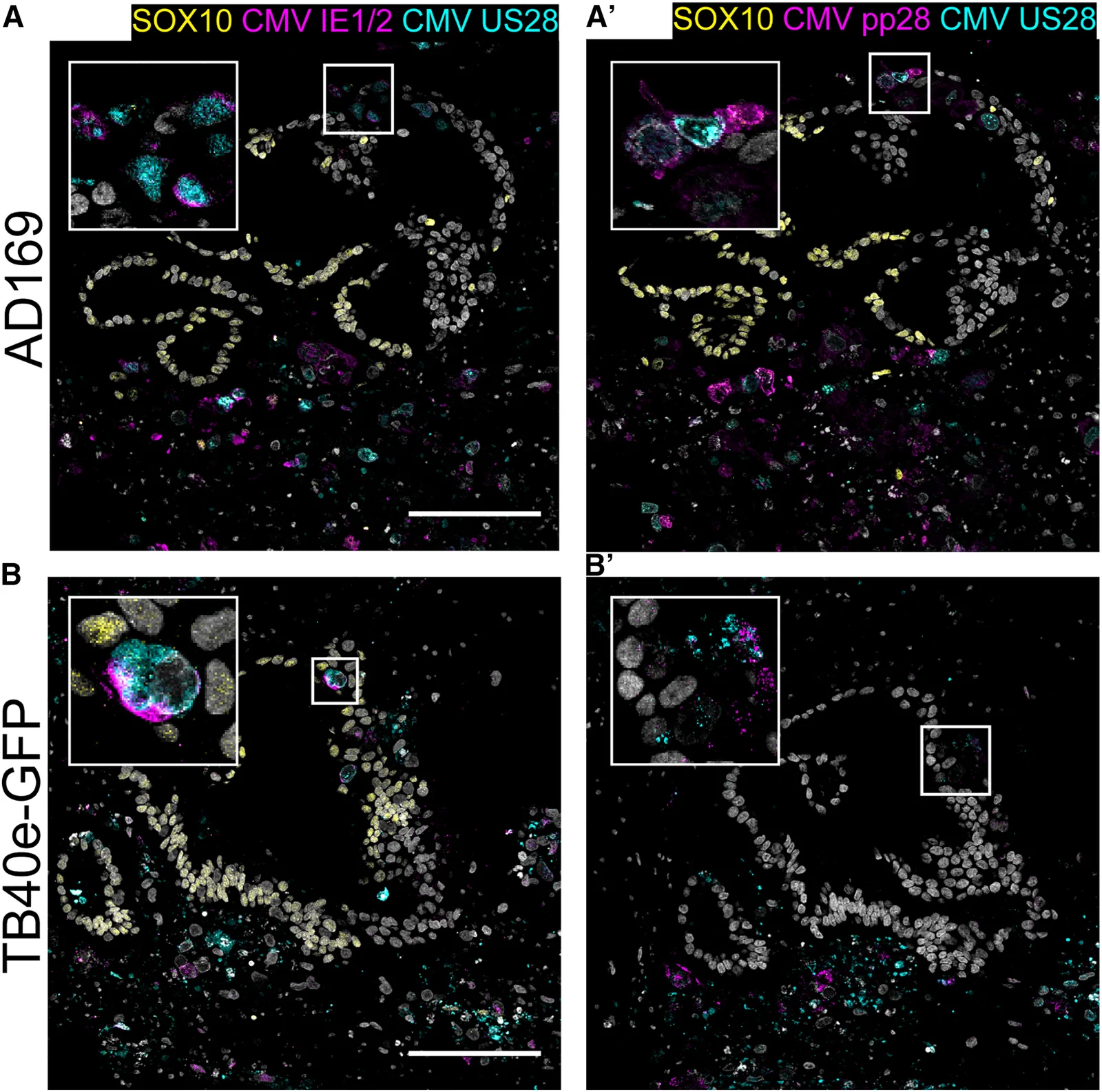
CMV strains complete the full replication cycle within the inner ear organoid Inner ear organoids were sectioned using a vibratome, inoculated with AD169, TB40e-GFP, and Clinical strain 1 and cultured for an additional 21 days. To determine whether the virus was replicating within the otic vesicles, the expression of viral proteins associated with different stages of the replication cycle was assessed. (A) Expression of CMV IE1/IE2 (an immediate-early protein, magenta) and CMV US28 (an early protein, cyan) in an otic vesicle inoculated with AD169. A magnification is shown in the white square. (A′) The same otic vesicle stained for CMVpp28 (a late protein, magenta) and CMVUS28 (cyan). (B) The same staining was performed on an otic vesicle inoculated with TB40e-GFP. Scale bar, 100 μm.

### HCMV laboratory strains predominantly infect nonsensory epithelial cells

To determine which inner ear cell types were affected following infection of the otic vesicles, immunofluorescent staining was performed on IEOs 21 days post-inoculation with AD169 and TB40e-GFP. Co-expression of CMV IE1/IE2 proteins was observed in SOX10-positive epithelial cells (Figures 4A–4C, 4F, 4G, and 4I), MYO7A-positive hair cells (Figures 4A, 4B, 4F, and 4G), and TUBB3-positive neurons that are in close proximity to otic vesicles (Figures 4D, 4E, and 4H; Table S1).

**Figure 4:**
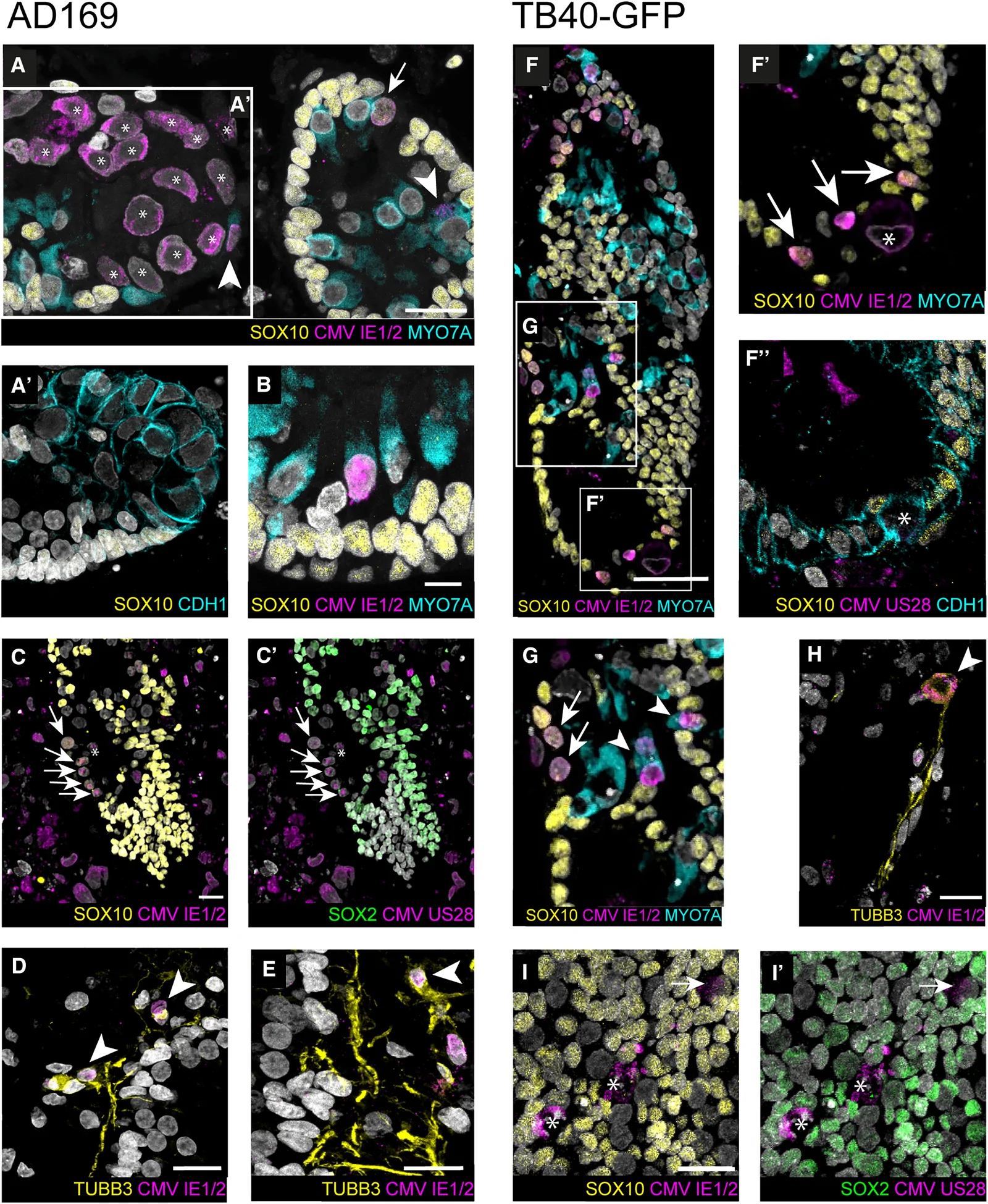
Laboratory strains infect nonsensory epithelial cells, hair cells, and neurons IHC of IEO slices inoculated with either AD169 (A–E) or TB40e-GFP (F–I) and cultured for 21 days. Infected epithelial cells (SOX10^+^, yellow) are indicated by arrows. Infected hair cells (MYO7A^+^, cyan) or neurons (TUBB3^+^, yellow) are indicated by arrowheads. Infected cells lacking co-expression with any otic marker are marked with an asterisk. (A) An epithelial cell and two hair cells infected with CMV. (A′) The indicated section stained for CDH1 (cyan), an epithelial cell marker that is not specific to the neural crest lineage. Scale bar, 50 μm. (B) Infected hair cell. Scale bar, 20 μm. (C) Infection is limited to nonsensory epithelial cells as sensory epithelium (SOX2^+^, green) is not affected by AD169. SOX10 staining is diminished in CMV IE1/IE2^+^ cells, but still present. Scale bar, 50 μm. (D) Infection of neurons. Scale bar, 50 μm. (E) Infection of a neuron. Scale bar, 100 μm. (F) Large otic vesicle infected by TB40e-GFP. Scale bar, 50. (F′) A higher-magnification image with infected epithelial cells and an infected unknown cell type. (F″) The same section stained for CDH1 (cyan). (G) Higher magnification of (F), showing infected epithelial cells and hair cells. (H) Infected neuron. Scale bar, 50 μm. (I) Staining for SOX10 and SOX2. (I′) An infected nonsensory epithelial cell (SOX10^+^, yellow). There is no co-expression of CMV US28 with SOX2. Scale bar, 20 μm.

Additionally, a subset of cells located within the otic vesicle showed CMV IE1/IE2^+^ expression and nuclear enlargement, but lacked expression of otic markers (SOX10, MYO7A, SOX2, and TUBB3) (Figures 4A–4C, 4F, and 4I). These cells retained CDH1 expression (Figures 4A′ and 4F″), suggesting epithelial origin despite lineage loss. Furthermore, CMV IE1/IE2^+^ cells were also detected outside the otic vesicle (Figures 2A, 2B, and 4C). These cells lacked OTOR, a marker for periotic mesenchyme (data not shown), and TUBB3 expression but, based on their location, are likely mesenchymal in identity.

Notably, no cells co-expressing CMV US28 protein and SOX2 were detected (Figures 4C′ and 4I′). CMV US28 is an early CMV protein. This absence could implicate that either SOX2-positive cells are not infected or CMV directly downregulates SOX2 expression.

Taken together, the results suggest that hCMV laboratory strains target not only nonsensory SOX10^+^SOX2^−^ epithelium but also hair cells and inner ear neurons. No clear differences were found between infection with AD169 and TB40e-GFP.

### Clinical hCMV strains infect similar otic cell types as laboratory strains

We next tested two patient-derived isolates (Clinical strain 1 and Clinical strain 2) to assess whether clinical strains infect similar cell types. Clinical strain 1 was isolated from the urine of a congenitally infected neonate, while Clinical strain 2 was obtained from the urine of an anonymous patient with PCR-confirmed hCMV infection. After four passages, Clinical strain 1 exhibited a substantially higher titer (1.3 × 10^6^ TCID_50_/mL) compared to Clinical strain 2 (1.1 × 10^3^ TCID_50_/mL).

Organoids exposed to Clinical strain 1 and cultured for 21 days showed expression of CMV IE1/IE2, US28, and pp28. As observed in laboratory strains, this pattern confirms progression through the different stages of the replication cycle of hCMV (Figure S3).

7 days post-inoculation with Clinical strain 1, IEOs displayed IE1/IE2^+^ clusters, expanding to the otic vesicle by day 21 (Figures 5A and 5B). Higher-magnification imaging revealed that CMV IE1/IE2^+^ cells within the otic vesicle did not co-express SOX10, although several cells (marked with an asterisk) likely originated from epithelial lineages based on their location within the otic vesicle (Figure 5C) and CDH1 expression (Figure 5E). In addition, infected hair cells (Figure 5D), neurons (Figure 5G), and SOX10-positive cells underneath the epithelium were observed (Figures 5B and 5C).

**Figure 5:**
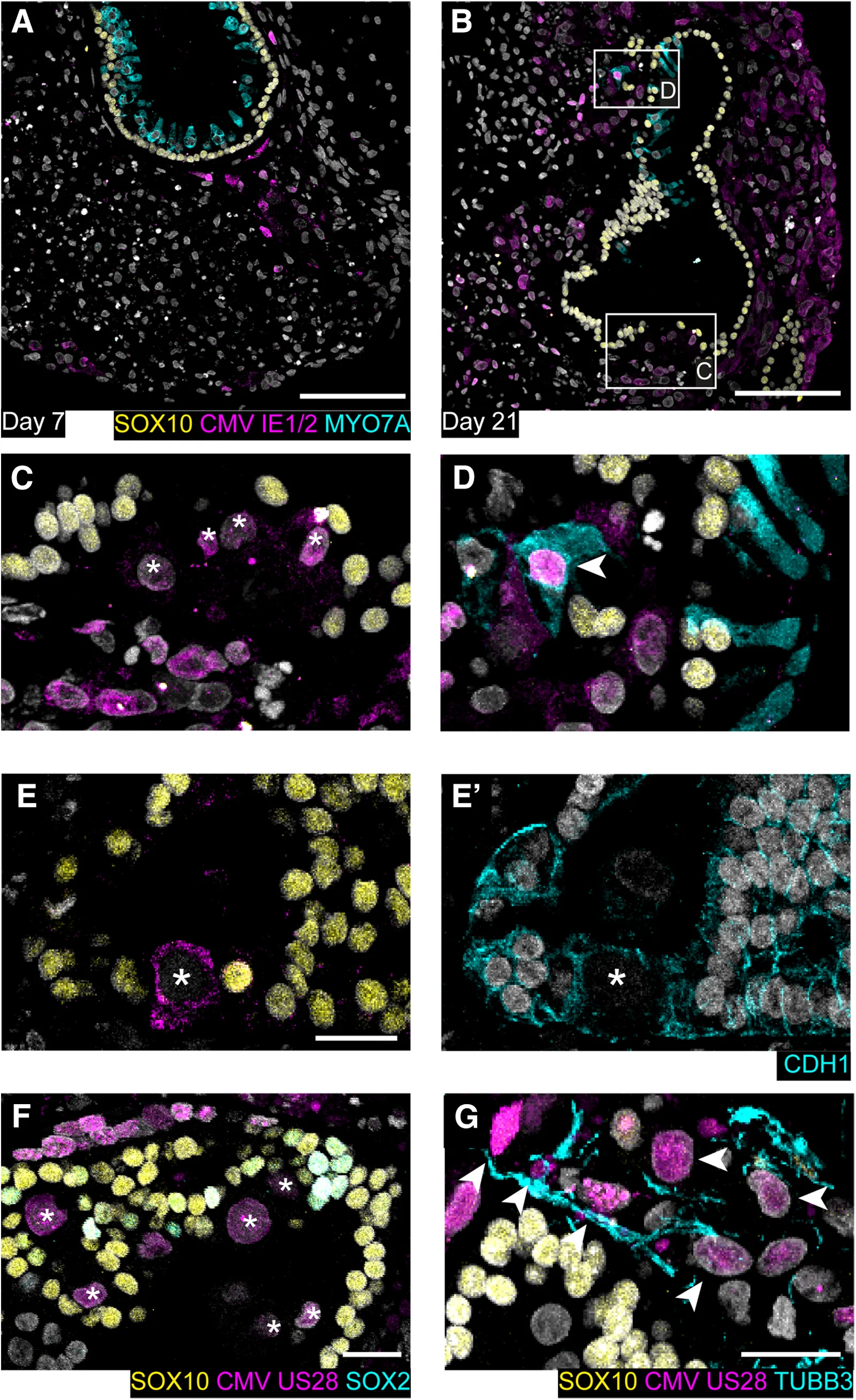
Clinical isolate infects epithelial cells, hair cells, and neurons Inner ear organoids were sectioned using a vibratome, inoculated with Clinical strain 1, and cultured for an additional (A) 7 or (B–G) 21 days. Epithelial cells are stained for SOX10 (yellow). Infected hair cells (MYO7A^+^, cyan) or neurons (TUBB3^+^, cyan) are indicated by arrowheads. Infected cells lacking co-expression with any otic marker are marked with an asterisk. (A) Infection of cells outside of the otic vesicle only after 7 days of culture. Scale bar, 100 μm. (B) Large otic vesicle infected by Clinical strain 1 after 21 days of culture. Scale bar, 100 μm. (C) Magnification of (B) showing infected cells lacking an otic marker. (D) Magnification of (B) showing an infected hair cell. (E) Infected cell lacking MYO7A and SOX10 expression, but showing CDH1 expression (E′). Scale bar, 20 μm. (F) No co-expression of CMV US28 with SOX2 or SOX10. Scale bar, 20 μm. (G) Infected neurons. Scale bar, 20 μm.

Co-expression of SOX2 and CMV US28 was absent (Figure 5F). Overall, the infection pattern mirrors what was observed when using laboratory strains.

Clinical strain 2 did not result in infected otic vesicle cells by day 7 post-inoculation. By day 21, most otic vesicles remained uninfected, with the exception of a single case where the vesicle was located near the periphery of the aggregate (Figures S4A, S4B, and S4C). In this instance, infected cells within the otic vesicle expressed CDH1 but lacked SOX10 and MYO7A, consistent with the infection pattern observed for the laboratory strains. To determine if this limited infection was due to the low titer of Clinical strain 2, we inoculated organoids with a dose equivalent of AD169 (5.5 × 10^2^ TCID_50_). Similar to the clinical strain, this low-dose AD169 failed to establish infection in the otic vesicles (Figures S4D and S4E) within 21 days, indicating that a minimum viral titer is required.

### CMV host receptors are expressed by otic cells within the organoid

CMV cell entry is mediated by several host receptors, including epidermal growth factor receptor (EGFR), neuropilin-2 (NRP2), and platelet-derived growth factor alpha (PDGFRA). To determine whether these receptors are present in the IEO, we analyzed single-nucleus RNA sequencing (snRNA-seq) data from the Human Inner Ear Atlas, derived from the same IEO protocol.^34^

EGFR transcripts were widely detected across multiple cell types, with elevated expression in periotic mesenchyme and hair cells (Figure S5A). This expression pattern was validated via IHC in day 75 organoids (Figure S5B), which corresponds to the stage used for inoculation. Specifically, EGFR protein was clearly localized to the mesenchyme and the stereocilia of the hair cells. NRP2 showed lower transcript levels, primarily localized to neurons, periotic mesenchyme, and hair cells (Figure S5A). IHC analysis revealed a punctate staining pattern throughout the epithelial and mesenchymal compartments (Figure S5C). Finally, PDGFRA expression was primarily restricted to mesenchymal cells, a finding confirmed by our IHC analysis (Figures S5A and S5C). Collectively, these data demonstrate that CMV host receptors are expressed in both periotic mesenchymal and epithelial cells, consistent with the observed infection of both cell types by laboratory and clinical CMV strains in this study.

## Discussion

This study presents the IEOs as a model for hCMV infection. We demonstrate that the virus infects the otic vesicles, alters the size of nuclei, and completes its full replication cycle within inner ear cells. Consistent with findings from postmortem analyses, infection affects multiple types of epithelial cells, neurons, and most likely mesenchymal cells.^19^^,^^20^^,^^21^^,^^22^^,^^23^

Co-localization of viral proteins and otic markers revealed infection of inner ear SOX10^+^SOX2^−^ epithelial cells, hair cells, and neurons, as well as CDH1^+^ epithelial cells lacking SOX10 expression. The latter may reflect downregulation of transcription factors upon infection. In neural progenitor cells, hCMV suppresses SOX2 expression within hours post-inoculation by targeting STAT3,^27^^,^^39^^,^^40^ a mechanism that could explain SOX2 loss in infected epithelial cells.

Our findings extend prior work in two directions. First, Harding et al. inoculated otic progenitor-like cells with hCMV after 20 days of differentiation.^41^ Although expression of certain otic markers was shown, the complex cellular composition of the neurosensory unit organization as in the IEO model, is lacking, hampering translatability. Second, the infection pattern we observed parallels postmortem reports of fetuses with congenital hCMV, where nonsensory epithelial cells are the predominant targets in both cochlear and vestibular organs, with occasional infection of hair cells and neurons.^19^^,^^20^^,^^21^^,^^22^^,^^23^^,^^24^ The detection of neuronal infection in our model (adjacent to but outside the otic vesicles) adds to inconsistent pathology reports and may represent a previously underappreciated route of virus-induced auditory or vestibular dysfunction.

CMV infection in the aforementioned cell types aligns well with our previous study, in which we observed the expression of hCMV receptors EGFR, NRP2, and PDGFRA in fetal tissue in mesenchymal cells, nonsensory epithelial cells, and stereocilia of hair cells.^42^ Our snRNA-seq and IHC analyses of the IEO revealed a highly conserved receptor expression pattern, further validating this organoid model. While our IHC analysis relied on anatomical localization rather than co-staining with cell-type-specific markers, the distinct cellular distribution of these receptors suggests a robust model for studying viral entry. Notably, the absence of macrophages in the current IEO platform precludes an assessment of their potential role in CMV receptor expression.

All viral strains tested, including the laboratory strains AD169 and TB40e-GFP and low-passage clinical isolates, infected otic vesicle epithelia with similar cell-type permissiveness. This was unexpected given AD169’s loss of the pentameric complex required for efficient epithelial infection. Residual epithelial infectivity of AD169 has been documented,^43^ and in our system, the use of high inoculum and extended culture may have compensated for reduced efficiency. The inclusion of low-passage clinical strains, which typically retain both trimeric and pentameric complexes and have broader tropism,^37^ enhances translational relevance. However, their use presents practical challenges: high viral titers are more difficult to achieve, as minimizing passage number is essential to preserve genetic integrity. The limited infection observed with Clinical strain 2 was likely due to lower titers, consistent with our finding that dose-matched inoculation with AD169 similarly failed to infect the otic vesicles. Additionally, the cell-to-cell spread characteristic of clinical isolates may further restrict their dissemination within the complex 3D architecture of the organoid compared to the more efficient cell-free spread of laboratory-adapted strains. We also cannot rule out the possibility that this specific isolate possesses lower intrinsic virulence or a slower replication kinetic in otic tissues.

In conclusion, IEOs offer a tractable human model to investigate hCMV pathogenesis in the developing inner ear, enabling studies that are not feasible *in vivo* or in animal models. Future directions include single-nucleus transcriptomic profiling to map infection-induced changes and the development of more consistent organoids to facilitate scalable, quantitative analysis of viral kinetics. Incorporation of protocols to generate cochlear sensory epithelium, stria vascularis, and immune components, alongside high-throughput screening for antiviral or otoprotective compounds, will expand the model’s fidelity and utility for elucidating mechanisms of virus-induced hearing and balance disorders.

### Limitations of the study

The current IEO model lacks several *in vivo* cell types potentially involved in hCMV pathogenesis, including the stria vascularis and dark cells as well as immune cells.^34^ The epithelia generated in this study are predominantly of vestibular identity, which still provides relevant insight given vestibular involvement in congenital hCMV, but restricts direct modeling of cochlear hair cell loss. Furthermore, the differentiation protocol employed in this study has been previously validated across eight distinct hPSC lines,^44^ demonstrating its robustness in generating inner ear tissues. To maintain experimental consistency for this foundational study, we utilized a single hiPSC donor line. However, we cannot rule out the possibility of a donor-specific effect on viral susceptibility or cellular diversity.

Otic vesicles are situated within a dense, multilineage cell aggregate dominated by mesenchyme and chondrocytes. While vascular endothelial cells have been identified within the organoids,^34^ the model lacks the extensive vascular network characteristic of the developing inner ear. To facilitate viral access to otic vesicles while preserving key aspects of the three-dimensional tissue architecture, the aggregates were sectioned using a vibratome. Although sectioning bypasses anatomical barriers and therefore does not fully recapitulate physiological viral entry or the sequence of viral dissemination *in vivo*, it allows us to evaluate the intrinsic susceptibility of these internal cell populations to direct viral exposure. This technique is widely accepted in organoid studies to bypass diffusion limits and prevent core hypoxia, and it has been validated within the IEO protocol.^44^^,^^45^^,^^46^^,^^47^ A previous study demonstrated that the structural and cellular integrity of otic vesicles are preserved 48 h post-sectioning.^46^ In our study, after 19 days of additional culture, CDH1^+^SOX10^+^ otic epithelium containing MYO7A hair cells and surrounding TUBB3^+^ neurons were present, indicating preservation of key anatomical features of the otic vesicles. However, whether functional properties are fully maintained across this 21-day post-sectioning period was not directly tested in the present study and remains to be evaluated.

## Resource availability

### Lead contact

Requests for further information and resources should be directed to and will be fulfilled by the lead contact, Lucia C.M. Grijpink (l.c.m.grijpink@lumc.nl).

### Materials availability

This study did not generate new unique reagents.

### Data and code availability

• All qPCR and IHC image analyses data generated for this study have been deposited at Mendeley Data: https://doi.org/10.17632/c2w9mrcmdr.1.
• This paper does not report original code
• Any additional information required to reanalyze the data reported in this paper are available from the lead contact upon request.

## Acknowledgments

The authors gratefully acknowledge Dr. Lance A. Mulder and Prof. Dasja Pajkrt, from the OrganoVIR Labs, Amsterdam UMC, for providing Clinical strain 1. We are thankful to the Facility for Light and Electron Microscopy of the Leiden University Medical Center for their support and assistance in this work. We also thank the Clinical Medical Microbiology Laboratory of the Leiden University Medical Center for their support with the qPCR experiments, in particular Dr. Els Wessels and Erin Meijers. This work was financially supported by the 10.13039/501100009708Novo Nordisk Foundation (NNF21CC0073729 to H.L.), 10.13039/501100022812Stichting Het Heinsius-Houbolt Fonds (H.L.), and 10.13039/501100003246NWO (NWA.1228.192.165 to A.C.T.M.V.).

## Author contributions

Conceptualization, H.L. and A.C.T.M.V.; data curation, L.C.M.G.; funding acquisition, H.L. and A.C.T.M.V.; investigation, L.C.M.G. and W.H.v.d.V.; methodology, L.C.M.G., A.C.T.M.V., H.L., W.H.v.d.V., and J.C.M.J.d.G.; resources, A.C.T.M.V., H.L., and W.H.v.d.V.; supervision, H.L. and A.C.T.M.V.; validation, L.C.M.G. and W.H.v.d.V.; visualization, L.C.M.G.; writing – original draft, L.C.M.G.; writing – review and editing, L.C.M.G., W.H.v.d.V., H.L., and A.C.T.M.V.

## Declaration of interests

W.H.v.d.V. and H.L., with the Leiden University Medical Center, have filed a patent application related to inner ear organoid differentiation used in this manuscript.

## Declaration of generative AI and AI-assisted technologies in the manuscript preparation process

During the preparation of this work, the authors used ChatGPT Plus in order to improve the readability and language of the article. After using this tool, the authors reviewed and edited the content as needed and take full responsibility for the content of the publication.

## STAR★Methods

### Key resources table

**Table:** 

REAGENT or RESOURCE	SOURCE	IDENTIFIER
**Antibodies**		

Mouse monoclonal anti-CDH1 (1:50)	BD Transduction Laboratories	Cat#: 610182; RRID: AB_397581
Mouse monoclonal anti-Cytomegalovirus IE1 and IE2 (1:100)	Abcam	Cat#: ab53495; RRID: AB_882995
Mouse monoclonal anti-Cytomegalovirus pp28 (1:1000)	Abcam	Cat#: ab6502; RRID: AB_305524
Rabbit polyclonal anti-Cytomegalovirus US28 (1:200)	Thermo Fisher Scientific	Cat#: PA5-102302; RRID: AB_2815916
Rabbit polyclonal anti-MYO7A (1:200)	Proteus Biosciences	Cat#: 25-6790; RRID: AB_10015251
Rabbit polyclonal anti-OTOR (1:100)	Invitrogen	Cat#: PA5-55053; RRID: AB_2645111
Mouse monoclonal anti-SOX2 (1:100)	BD Biosciences	Cat#: 561469; RRID: AB_10694256
Goat polyclonal anti-SOX10 (1:50)	Invitrogen	Cat#: PA5-47001; RRID: AB_2608449
Goat polyclonal anti-SOX10 (1:200)	R&D Systems	Cat#: AF2864; RRID: AB_442208
Mouse monoclonal anti-TUBB3 (1:100)	R&D Systems	Cat#: MAB1195; RRID: AB_357520
Rabbit polyclonal anti-TUBB3 (1:300)	Abcam	Cat#: ab18207; RRID: AB_444319
Donkey anti-mouse Alexa Fluor™ 488 (1:500)	Invitrogen	Cat#: A21202; RRID: AB_141607
Donkey anti-mouse Alexa Fluor™ 594 (1:500)	Invitrogen	Cat#: A21203; RRID: AB_141633
Donkey anti-rabbit Alexa Fluor™ 488 (1:500)	Invitrogen	Cat#: A21206; RRID: AB_2535792
Donkey anti-rabbit Alexa Fluor™ 594 (1:500)	Invitrogen	Cat#: A21207; RRID: AB_141637
Donkey anti-goat Alexa Fluor™ 680 (1:500)	Invitrogen	Cat#: A21084; RRID: AB_2535741
DAPI	Invitrogen	Cat#: D3571; RRID: AB_2307445
Goat IgG isotype control (1:1250)	Novus Biologicals	Cat#: NB410-28088; RRID: AB_1853319
Mouse IgG isotype control (undiluted)	Invitrogen	#Cat: 08-6599; RRID: AB_2532952
Rabbit IgG isotype control (undiluted)	Invitrogen	#Cat: 08-6199; RRID: AB_2532942

**Bacterial and virus strains**		

AD-169	ATCC	Cat#: VR-538
TB40e-delUL16-GFP	Prof. Dr. Christian Sinzger, Prof. Emmanuel J.H.J. Wiertz and Dr. Robert Jan Lebbink	N/A
CMV653941 (Clinical strain 1)	Prof. Dasja Pajkrt, Dr. Lance Mulder, OrganoVIR Labs	N/A
Clinical strain 2	This paper	N/A

**Chemicals, peptides, and recombinant proteins**		

Fetal bovine serum	Capricon Scientific	Cat#: CP18-2134
Minimum essential medium	Gibco	Cat#: 21090-022
Minimum essential medium, HEPES	Gibco	Cat#: 12360038
L-glutamin	Thermo Fisher Scientific	Cat#: A2916801
MEM nonessential amino acid solution	Thermo Fisher Scientific	Cat#: 11140050
HEPES	Thermo Fisher Scientific	Cat#: 15630106
Sodium pyruvate	Gibco	Cat#: 11360039
Amphotericin B	Gibco	Cat#: 15290018
Vancomycin	Hikma Pharma Benelux BV	Cat#: 16754891
Penicillin-streptomycin	Thermo Fisher Scientific	Cat#: 15070063
Gentamycin	Gibco	Cat#: 15750-037
Low melting point agarose	Sigma-Aldrich	Cat#: A0701
Histogel	Epredia	Cat#: HG-4000-012
Hematoxylin	Klinipath	Cat#: 4085-9001
Eosin	Klinipath	Cat#: 4082-9002
Bovine serum albumin	Sigma-Aldrich	Cat#: A3059-50G
Tween 20	Merck Group	Cat#: 8.22184
Prolong Gold antifade reagent	Invitrogen	Cat#: P36934
Trypsin-EDTA (0.05%), phenol red	Gibco	Cat#: 25300062
Avicel RC-581	FMC BioPolymer	N/A
Essential 8 Flex medium	Gibco	Cat#: A2858501
Vitronectin	Gibco	Cat#: A14700
Normocin	Invivogen	Cat#: ant-nr-1
EDTA	Gibco	Cat#: 15575020
Stempro Accutase Cell Dissociation Reagent	Gibco	Cat#: A1110501
mTeSR™ Plus	Stemcell Technologies	Cat#: 10027
Chroman 1	MedChem Express	Cat#: HY-15392
Emricason	MedChem Express	Cat#: HY-10396
Polyamine Supplement	Sigma-Aldrich	Cat#: P8483-5ML
transISRIB	Tocris	Cat#: 5248
E6 medium	Gibco	Cat#: A1516401
Matrigel Growth Factor Reduced	Corning	Cat#: 354230
SB431542	Stemgent	Cat#: 04-0010-05
basic FGF	PeproTech	Cat#: 100-18B
BMP-4	R&D Systems	Cat#: 314-BPE
LDN	Stemgent	Cat#: 04-0074-02
CHIR99021	Stemgent	Cat#: 04-0004-02
DMEM:F12	Gibco	Cat#: 12634010
Neurobasal Medium	Gibco	Cat#: 21103049
Vitamin A	Gibco	Cat#: 12587010
N2 supplement	Gibco	Cat#: 17502048
Glutamax	Gibco	Cat#: 35050061
β-Mercaptoethanol	Gibco	Cat#: 21985023
HotStarTaq Master Mix	Qiagen	Cat#: 203443

**Deposited data**		

qPCR data and IHC analyses data	This paper, Mendeley data	Mendeley data: https://doi.org/10.17632/c2w9mrcmdr.1

**Experimental models: Cell lines**		

MRC5-cells	ATCC	Cat#: CCL-171; RRID: CVCL_0440
LUMC0004iCtrl10 iPSC line	LUMC iPSC Hotel	N/A

**Software and algorithms**		

Las X Life Science	Leica	https://www.leica-microsystems.com/
ImageJ-Fiji	ImageJ	https://imagej.net/software/fiji/
SlideViewer	3DHistech	https://partners.3dhistech.com/software-downloads/?e-filter-9011b9a-downloads_cat=windows
IBM SPSS statistics for Windows version 29.0.0.0 (241)	IBM	N/A
Adobe Illustrator 2022	Adobe	https://www.adobe.com/nl/
Human inner ear atlas	gEAR: Gene Expression Analysis Resource	https://umgear.org/p?l=hIEOandInnerEar

**Other**		

96-well U-bottom plates with a super-low cell attachment surface	Thermo Fisher Scientific	Cat#: 174925
24-well plates with a super-low cell attachment surface	Thermo Scientific	Cat#: 174930
MagNA Pure 96 DNA and Viral NA Small Volume Kit	Roche Diagnostics	Cat#: 06543588001
CFX96 Touch Real-Time PCR Detection System	Bio-Rad	Cat#: 1855196

### Experimental model and study participant details

#### Cell line

MRC-5 cells were purchased directly from the American Type Culture Collection (ATCC, CCL-171). Cells were authenticated by the vendor prior to purchase and were not independently authenticated in-house. MRC-5 cells were cultured in minimum essential medium (Gibco) supplemented with 9% fetal bovine serum (Capricorn Scientific), 0.9% Amphotericin-B (Gibco), 0.9% L-Glutamin (Thermo Fisher Scientific), 0.1% Gentamycin (Gibco), 0.9% MEM Nonessential amino acid solution (Thermo Fisher Scientific) and 0.9% sodium pyruvate (Gibco). Passage numbers 14-23 were used.Mycoplasma contamination testing was conducted periodically throughout the study, and all cultures remained negative.

Organoids were generated from a healthy in-house derived human induced pluripotent stem cell (hiPSC) line. The cell line (LUMC004iCtrl10; male, ethnicity unknown; passages 20–26), was produced by the Human iPSC Hotel at Leiden University Medical Center. Prior to use, the line was confirmed to be free of mycoplasma contamination, exhibited a normal karyotype, and was validated for pluripotency. Cells were cultured on 6-well plates coated with recombinant human vitronectin (Gibco, A14700; 0.5 mg/cm^2^) in Essential 8 Flex medium (Gibco, A2858501) supplemented with Normocin (Invivogen, ant-nr-1; 100 mg/mL), hereafter referred to as E8 medium. Medium was refreshed daily or every other day depending on cell confluency. Cells were passaged approximately every 4–5 days at ∼80% confluency, using 0.5 mM EDTA (Gibco, 15575020; stock concentration 10 mM), and replated as small clusters of 3–5 cells.

#### Viral strains

TB40/E-delUL16-GFP (TB40e-GFP) was generated from TB40/E as described earlier^48^ and was kindly provided by Prof. Christian Sinzger, Institute of Virology, Ulm University Medical Center, and by Prof. Emmanuel J.H.J. Wiertz and Dr. Robert Jan Lebbink, UMC Utrecht. TB40e-GFP and AD169 were propagated in MRC-5 cells for 2-4 passages in MEM containing HEPES (Gibco), supplemented with 4.6% fetal bovine serum (Capricorn Scientific), 0.9% amphotericin B (Gibco), 0.9% L-glutamin (Thermo Fisher Scientific), 0.1% gentamycin (Gibco), 0.9% MEM nonessential amino acid solution (Thermo Fisher Scientific) 0.9% sodium pyruvate (Gibco) and 0.1% vancomycin (Hikma Pharma Benelux BV).

Clinical strain CMV653941 (referred to hereafter as Clinical strain 1) was kindly provided by Prof. Dasja Pajkrt and Dr. Lance Mulder from the OrganoVIR Labs, Amsterdam UMC. This strain was obtained from urine of a 3 days old patient with congenital CMV infection with severe neurological sequelae, including sensorineural hearing loss and was propagated as previously described.^26^

Clinical strain 2 was derived from an anonymous patient with active CMV infection, confirmed by PCR on plasma. A urine sample was collected and diluted 1:1 in culture medium (identical to the medium used for TB40e-GFP and AD169 propagation), then passed through a 0.45 μm filter. A total of 330 μL diluted urine sample was added to each well of a 24-well plate containing a confluent monolayer of MRC-5 cells. The plate was centrifuged at 300 × g for 30 min, followed by incubation for 45 min at 37 °C with 5% CO_2_. After incubation, the inoculum was removed, cells were washed with medium, and 1 mL fresh medium was added per well. Cells were incubated at 37 °C, 5% CO_2_ until cytopathic effects became apparent (∼2 weeks post-inoculation).

Cells were then trypsinized and transferred to a T25 flask containing confluent MRC-5 cells. After 15 days, the culture was expanded to a T75 flask, and subsequently to two T75 flasks. Once cytopathic effect reached approximately 90%, the viral stock was harvested and frozen, as described below.

The supernatant was centrifuged at 3,500 rpm for 5 min. Cells in the T75 flask were trypsinized and pelleted at 1,000 rpm for 5 min. Both pellets were pooled and resuspended in culture medium. Two freeze–thaw cycles were performed using a –80 °C freezer and a 37 °C water bath. The suspension was clarified by centrifugation at 3,500 rpm for 8 min, and the supernatant was mixed 1:1 with 50% sorbitol in Milli-Q water. The final viral stock was stored at –80 °C. Viral titer was determined by TCID_50_ assay.

### Method details

#### Inner ear organoid differentiation protocol

Inner ear organoids were generated following established protocols with minor adjustments.^34^^,^^44^^,^^49^ Briefly, hiPSC colonies were dissociated into single cells using Accutase (Gibco, A1110501) and suspended in mTeSR medium (Stemcell Technologies, 100-0276) supplemented with CEPT Cocktail,^50^ containing 50 nM Chroman 1 (MedChem Express, HY-15392), 5 μM Emricasan (MedChem Express, HY-10396), 1X Polyamine Supplement (Sigma-Aldrich, P8483-5ML), and 700 nM trans-ISRIB (Tocris, 5284). A total of 2,500 cells in 100 μL of this suspension were seeded into 96-well U-bottom plates with a super-low attachment surface (Thermo Scientific, 174925), centrifuged at 110 × g for 6 minutes, and incubated at 37°C in 5% CO_2_ for 48 hours.

On day 0 (D0), cell aggregates were transferred to E6 medium (Gibco, A1516401) containing Normocin (100 μg/mL), 2% Matrigel Growth Factor Reduced (Corning, 354230), 10 μM SB431542 (Stemgent, 04-0010-05), 4 ng/mL basic FGF (bFGF; PeproTech, 100-18B), and 5–15 ng/mL BMP-4 (R&D Systems, 314-BPE). On day 3 (D3), 25 μL of E6 medium with 200 ng/mL LDN (Stemgent, 04-0074-02) and 50 ng/mL bFGF was added, increasing the total volume to 125 μL. On day 6 (D6), the medium was topped up to 200 μL with fresh E6. On day 8 (D8), 100 μL of medium was replaced with E6 containing 6 μM CHIR99021 (CHIR; Stemgent, 04-0004-02) to induce otic placode formation. On day 10 (D10), 100 μL of medium was replaced with E6 containing 3 μM CHIR. On day 12 (D12), aggregates were transferred to Organoid Maturation Medium (OMM) in 24-well low-attachment plates (Thermo Scientific, 174930) and maintained on an orbital shaker at 37°C. OMM consisted of a 1:1 mixture of advanced DMEM:F12 (Gibco, 12634010) and Neurobasal Medium (Gibco, 21103049), supplemented with 0.5× B27 without vitamin A (Gibco, 12587010), 0.5× N2 Supplement (Gibco, 17502048), 1× Glutamax (Gibco, 35050061), 0.1 mM β-mercaptoethanol (Gibco, 21985023), Normocin (100 μg/mL), and 1% Matrigel with 3 μM CHIR.

On day 15 (D15), a half medium change was performed with OMM containing 1% Matrigel and 3 μM CHIR. On day 18 (D18), another half medium change with OMM was done, diluting Matrigel and CHIR. On day 21 (D21), a full medium change with fresh OMM was conducted. From day 21 onward, medium changes followed a 7-day schedule: half the medium was replaced on days 0 and 2, with a full change on day 4. Starting from day 30, Amphotericin B (Gibco, 15-290-018) was added to the medium. Medium volume was gradually increased, 500 μL per well on day 12, 1 mL by day 45, and 1.5 mL by day 60.

#### Plaque assay

AD169 and TB40e-GFP were quantified using a plaque assay. Viral stocks were 10-fold serially diluted in medium. Confluent MRC-5 cell monolayers in a 6-well plate were inoculated with 500 μL of diluted virus per well and incubated for 2.5 h at 37 °C, 5% CO_2_, with regular gentle shaking. After incubation, the inoculum was removed and replaced with 5 mL of an overlay, composed 42.5% sterile-filtered 2× DMEM, 2% fetal bovine serum, 0.5% penicillin–streptomycin (Thermo Fisher Scientific), 5% 1 M HEPES (Thermo Fisher Scientific), and 50% 2.4% Avicel. Cells were incubated for 14 days at 37 °C, 5% CO_2_. After incubation, wells were washed with PBS and fixed with 4% formaldehyde. Plaques were visualized by crystal violet staining.

#### TCID50 assay

Clinical strains were titrated using TCID_50_ assays based on the Reed–Muench method.^51^ Clinical strain 1 was titrated on ARPE-19 cells, and infectious titers were determined after 17 days. Clinical strain 2 was titrated on MRC-5 cells, with titers determined after 13 days. Each viral strain was tested in triplicate.

Viral stocks of clinical strains were titrated after their use in infection experiments with inner ear organoids (IEOs); as a result, titers could not be directly compared across different experiments or between strains. Due to the three-dimensional architecture and heterogeneous cell composition of the IEOs, multiplicity of infection (MOI) was not calculated. Instead, the TCID_50_ per mL of each viral stock used is reported.

#### Vibratome sectioning and viral inoculation of inner ear organoid aggregates

On day 75 of IEO culture, 3–4 aggregates were pooled into a single block using 4% low-melting-point agarose in PBS (Sigma-Aldrich) and sectioned at 300 μm thickness using a vibratome (Leica VT1200S) set to amplitude 1 and a speed of 0.2 mm/s. Sections were collected in ice-cold PBS, and bright-field evaluation was performed using a Leica IC90 E microscope to identify and select sections containing otic vesicles.

Selected vibratome sections were transferred to OMM and incubated overnight at 37 °C with 5% CO_2_. Due to lower viral titer of the clinical isolates, infections with clinical strains were performed in 6-well plates containing 2 vibratome slices, while those with laboratory-adapted strains were carried out in 24-well plates containing 1 vibratome slice. The following day, sections were inoculated with either 10^4^ plaque forming units (PFU) of AD169 (stock concentration: 10^3^ – 10^5^ PFU/ml) or TB40e-GFP (stock concentration: 10^4^ PFU/mL). For clinical strains, where plaque formation was not observed, titers were determined via TCID_50_ assay. Sections were inoculated with 1.4 × 10^5^ TCID_50_ of Clinical Strain 1 (stock concentration: 1.3 x 10^6^ TCID_50_/mL), or 5.5 × 10^2^ TCID_50_ of Clinical Strain 2 (stock concentration: 1.1 x 10^3^ TCID_50_/mL). To directly compare viral kinetics at a lower multiplicity of infection, an additional cohort was inoculated with a dose-equivalent of AD169 (5.5 x 10^2^ TCID_50_), calculated from an empirical TCID_50_ titration of the AD169 stock (6.3 10^6^ TCID_50_/ml). Negative controls included sections cultured in OMM alone or exposed to heat-inactivated virus, prepared by incubating viral stocks at 60 °C for 30 minutes.

The inoculum was left on overnight. Subsequently, sections were washed with PBS, and fresh OMM was added. Every 3 days, a sample of the culture supernatant was collected for CMV DNA PCR analysis, and the medium was replaced. After 7 or 21 days, the vibratome sections were washed with PBS and fixed in 4% buffered formaldehyde for 2 h at room temperature.

#### Tissue section preparation and immunohistochemical staining

Histogel (Epredia) was used to embed 3–4 aggregate slices in the same horizontal plane. After dehydration through an ascending ethanol series (70–99%) and clearing in xylene, tissues were embedded in paraffin and sectioned at 5 μm using a rotary microtome (Thermo Fisher Scientific, HM 355 S). Sections were mounted on silane-coated glass slides and air-dried overnight at room temperature.

Tissue sections were deparaffinized in xylene and rehydrated through a descending ethanol series (96%–70%–50%). Routine hematoxylin and eosin (H&E) staining was performed on every fifth section to identify anatomical regions and relevant cell types (Klinipath).

For immunohistochemistry (IHC), heat-induced antigen retrieval was performed in 10 mM sodium citrate buffer (pH 6.0, adjusted with hydrochloric acid). To block nonspecific binding, sections were incubated for 30 min in blocking buffer (5% bovine serum albumin (Sigma-Aldrich), 0.05% Tween-20 (Merck Group) in PBS). Sections were then incubated overnight at 4 °C with primary antibodies or corresponding isotype controls.

A second blocking step was applied for 10 min, followed by incubation with secondary antibodies and DAPI (Invitrogen) for 1 h at room temperature. Washing steps between incubations were performed with 0.05% Tween-20 in PBS. Slides were mounted using ProLong Gold Antifade Reagent (Invitrogen) and coverslipped. For slides exposed to TB40e-GFP, CMV proteins were consistently imaged using the 488-nm channel to ensure that GFP fluorescence was not misinterpreted as signal from other targets.

#### Imaging and interpretation

GFP signal during culture with the TB40e-GFP CMV strain was imaged using an EVOS FL Digital Inverted Microscope.

H&E-stained sections were scanned using a 3DHISTECH Pannoramic 250 slide scanner and viewed with SlideViewer software (3DHISTECH). Immunostained sections were imaged using a Leica SP8 confocal laser scanning microscope and a Leica DM5500 B fluorescence microscope, operating under Leica Application Suite X (LAS X) software (Leica Microsystems).

Maximal intensity projections were generated from confocal image stacks. Fiji (ImageJ version 1.53c) was used to adjust brightness and contrast and to perform nuclear counts and sizing.

The percentage of infected cells was determined from immunofluorescence images acquired using a Leica DM5500 B fluorescence microscope with a 10× objective. Infected nuclei were identified by comparing DAPI staining to CMV IE1/IE2 immunolabeling, which both exhibit nuclear signal. Nuclei were counted by applying a consistent threshold and analyzing particles with a minimum area of 5 μm^2^. The proportion of infected nuclei was determined by dividing the number of infected nuclei by all DAPI stained nuclei. At least three images were analyzed per condition, each image taken at the edge of the aggregate.

Nucleus size was manually measured using the freehand selection tool in ImageJ. 23 CMV IE1/IE2^+^ nuclei were compared to 91 CMVIE1/IE2^-^ nuclei.

#### Quantitative PCR

Starting at day 1 post-inoculation, 220 μL of supernatant was collected every 3 days, prior to medium change, and stored at –80 °C. For quantification of CMV DNA, the LDT CMV assay was used, which is based on the previously published PCR assay utilizing primers and probes to amplify a 126-bp fragment from the CMV immediate-early antigen region, with minor modifications.^52^ DNA isolation was performed using the MagNA Pure 96 platform with the DNA and Viral NA Small Volume Kit 2.0 using the Pathogen Universal 200 protocol (Roche Diagnostics). The PCR reaction was carried out using the HotStarTaq Master Mix (Qiagen) in the CFX96 Touch Real-Time PCR Detection System (Bio-Rad). The LDT CMV assay has been used in routine clinical practice for nearly 20 years, is standardized to the first WHO International Standard,^53^ and quantifies results between 1.7 and 7.0 Log IU/mL. To ensure measurements remained within this range, samples were diluted in PBS as needed.

#### Single nucleus RNA sequencing

To characterize host receptor expression within the IEO, we utilized publicly available snRNA-seq data from the Human Inner Ear Atlas, accessible via the Gene Expression Analysis Resource (https://umgear.org/p?l=hIEOandInnerEar). This dataset was generated using the same IEO differentiation protocol employed in the current study, ensuring methodological consistency. A detailed description of the dataset generation and primary analysis was previously published.^34^

### Quantification and statistical analysis

Each culturing condition was tested in 6-8 vibratome sections per experiment. Inoculation with AD169 was also replicated in a separate experiment. Immunohistochemical staining was conducted on at least two levels per paraffin block. The images presented in this article are representative of the combined results from these experiments.

To compare viral load over time across strains, Generalized Estimating Equation (GEE) was used.

The percentage of CMV-infected nuclei was assessed using the two-way ANOVA. Because normality was not met for some groups, we confirmed key findings using Mann–Whitney U tests, which yielded consistent results (TB40e-GFP day 7 vs 21, *p* = 0.027; TB40e-GFP vs mock on day 21, *p* = 0.014; AD169 vs mock on day 21, *p* = 0.003).

The difference in nucleus size between infected and non-infected cell was assessed using the Mann–Whitney U test.

All statistical tests were performed using IBM SPSS Statistics version 29.0.0.0 (241).
