# Supplementary material for: Modeling congenital cytomegalovirus infection in human inner ear organoids
Source: iScience. 2026 Nov 6;29(11):118148. doi: 10.1016/j.isci.2026.118148 (PMC13760963; doi:10.1016/j.isci.2026.118148)
Supplement: Supplementary file 1 [file mmc1.pdf]

iScience, Volume *29*

Supplemental information

Modeling congenital cytomegalovirus infection in human inner ear organoids

Lucia C.M. Grijpink, Wouter H. van der Valk, John C.M.J. de Groot, Heiko Locher, and Ann C.T.M. Vossen

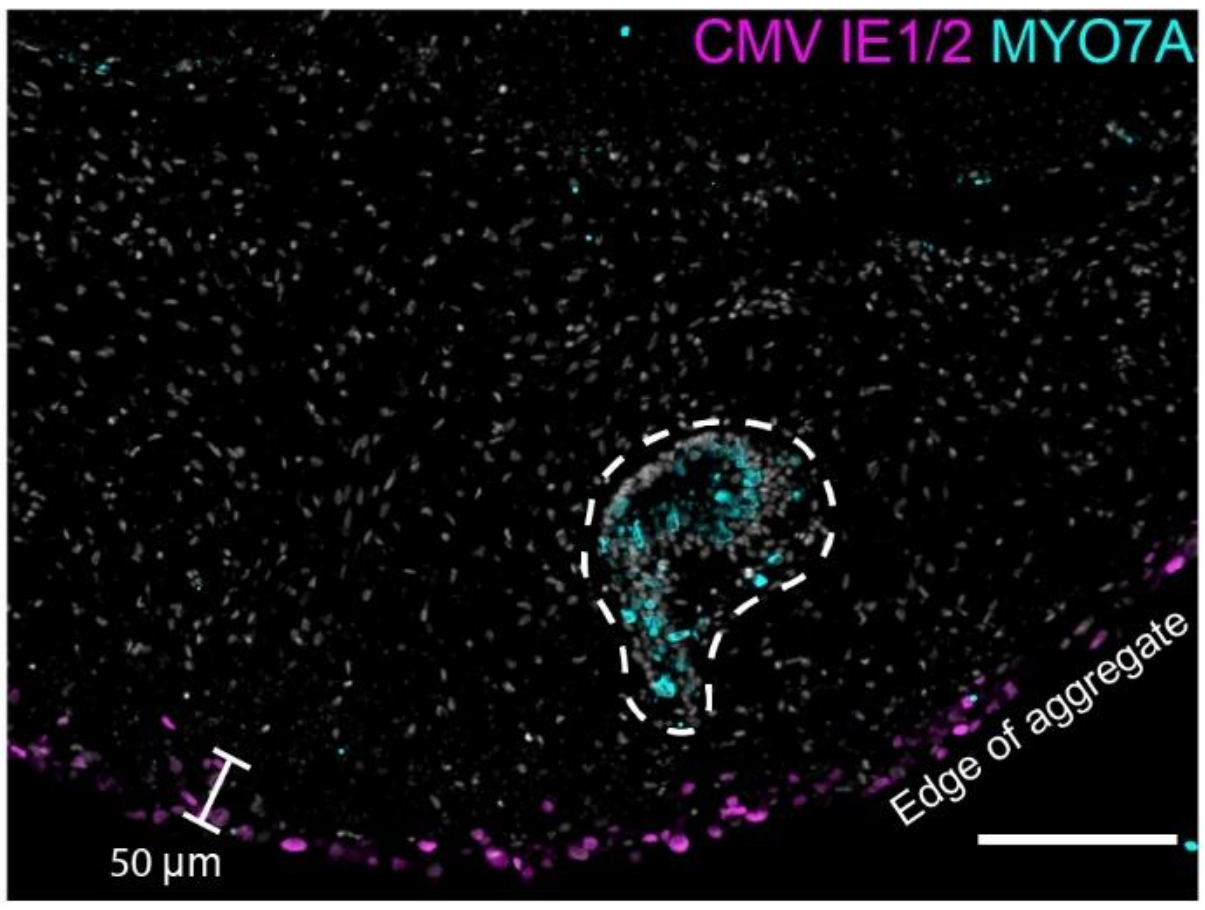

Figure S1. Viral penetration requires sectioning of the aggregate

An unsectioned inner ear organoid aggregate (day 75) was inoculated with AD169 and cultured for an additional 20 days. Immunostaining for CMV IE1/IE2 proteins (magenta) revealed signal restricted to the periphery of the aggregate. The otic vesicle, outlined with a dotted line, contains hair cells (MYO7A, cyan). Scale bar: 150 µm.

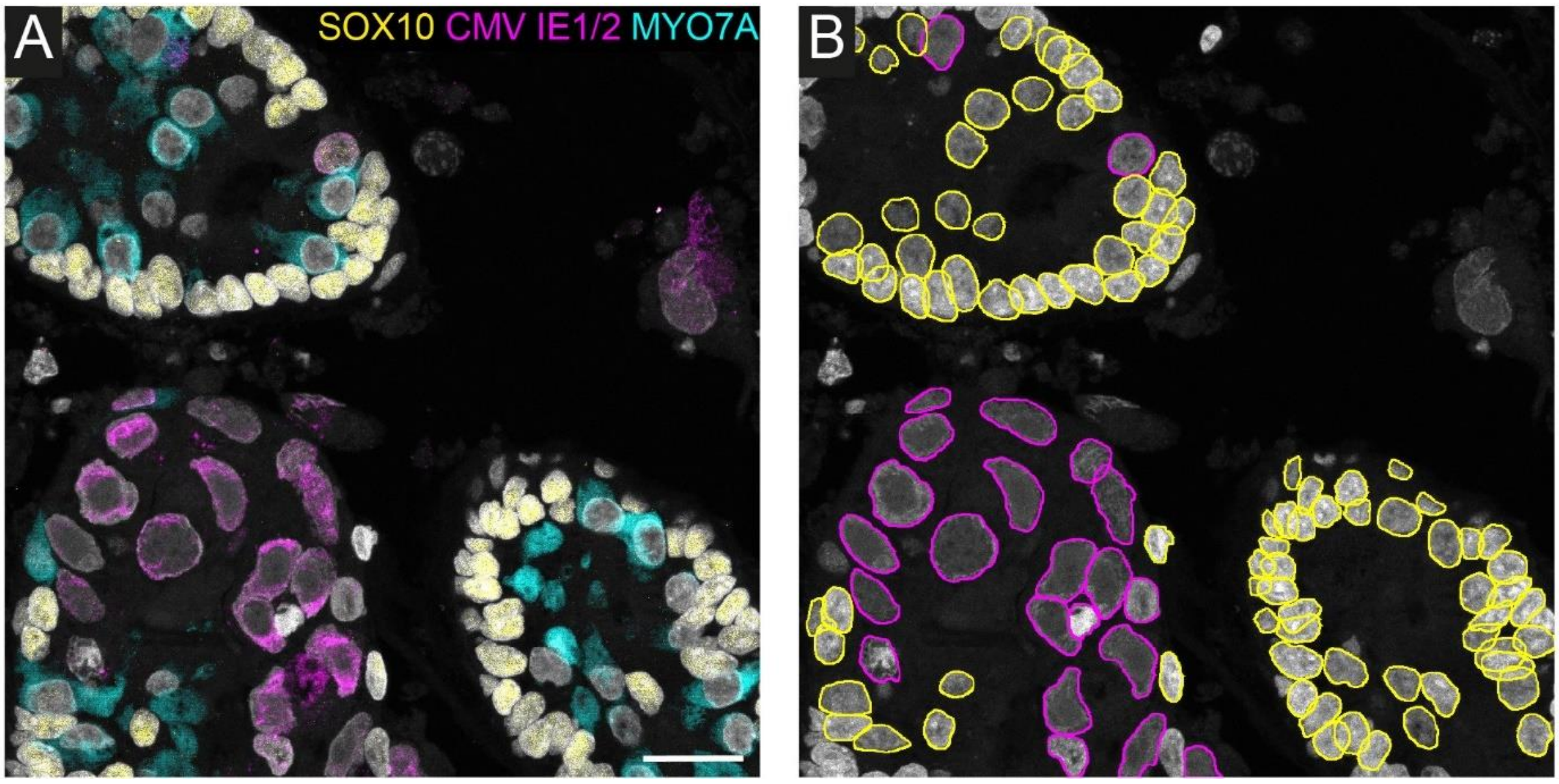

Figure S2. AD169-infected otic cells show nuclear enlargement

An IEO slice was inoculated with AD169 and cultured for 21 days.

(A) Immunohistochemistry image of an otic vesicle showing nuclei (DAPI, grey), epithelial cells (SOX10, yellow), hair cells (MYO7A, cyan), and CMV-infected cells (CMV IE1/IE2, magenta). Scale bar: 50 µm. CMV IE1/IE2 and DAPI signals were used to guide nuclear segmentation shown in (B).

(B) Otic nuclei were manually outlined and categorized as CMV^+ (magenta) or CMV^- (yellow) based on IE1/IE2 expression. Quantification of nuclear size is presented in Figure 2E.

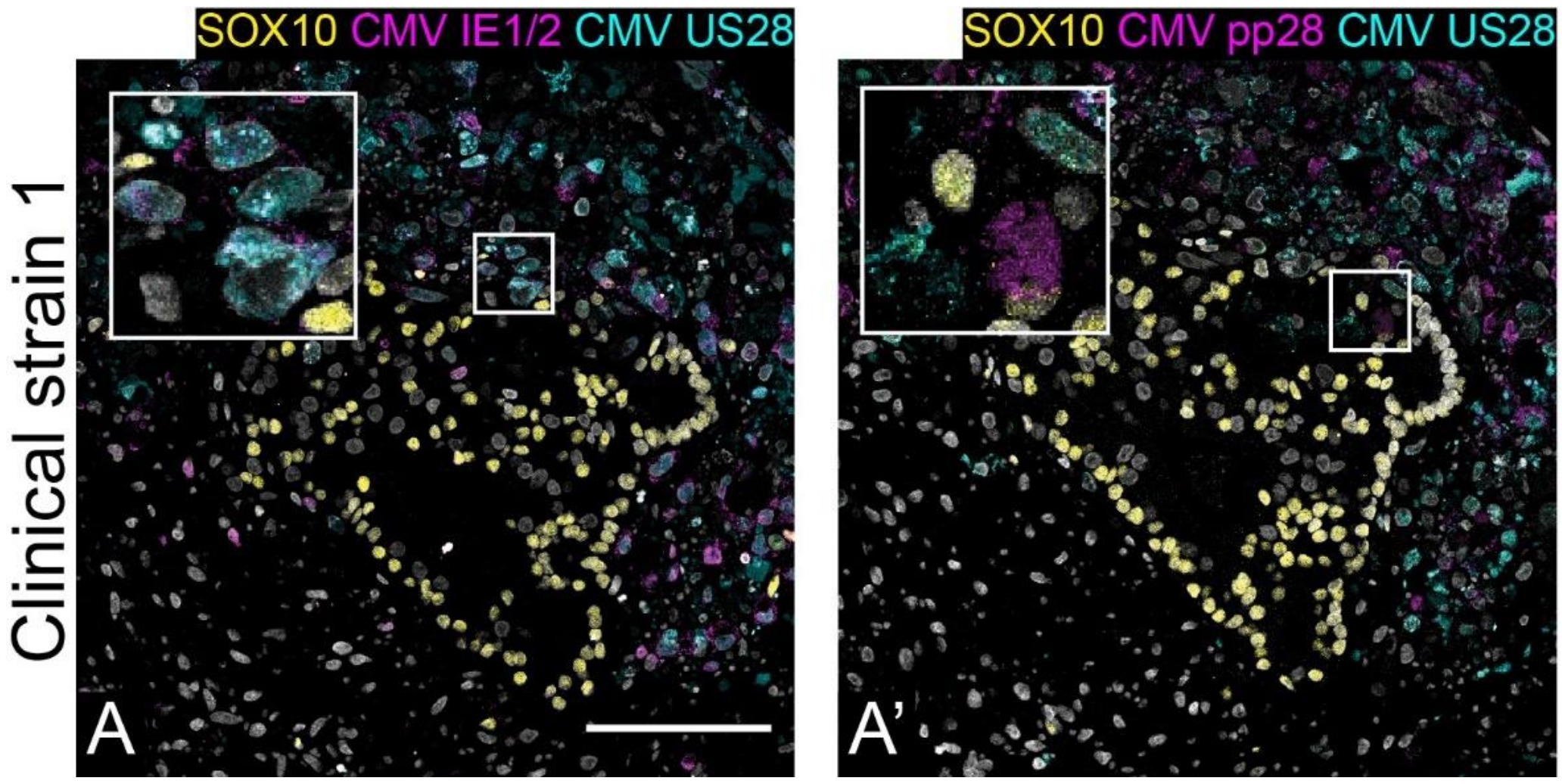

Figure S3. **Clinical strain 1 completes the full replication cycle within the inner ear organoid**

Inner ear organoids were sectioned using a vibratome, inoculated with Clinical strain 1 and cultured for an additional 21 days. To determine whether the virus was replicating within the otic vesicles, the expression of viral proteins associated with different stages of the replication cycle was assessed.

(A) Expression of CMV IE1/IE2 (an immediate early protein, magenta) and CMV US28 (an early protein, cyan) in an otic vesicle inoculated with Clinical strain 1. A magnification is shown in the white square. A’ shows the same otic vesicle stained for CMVpp28 (a late protein, magenta) and CMVUS28 (cyan). Scale bar = 100 µm

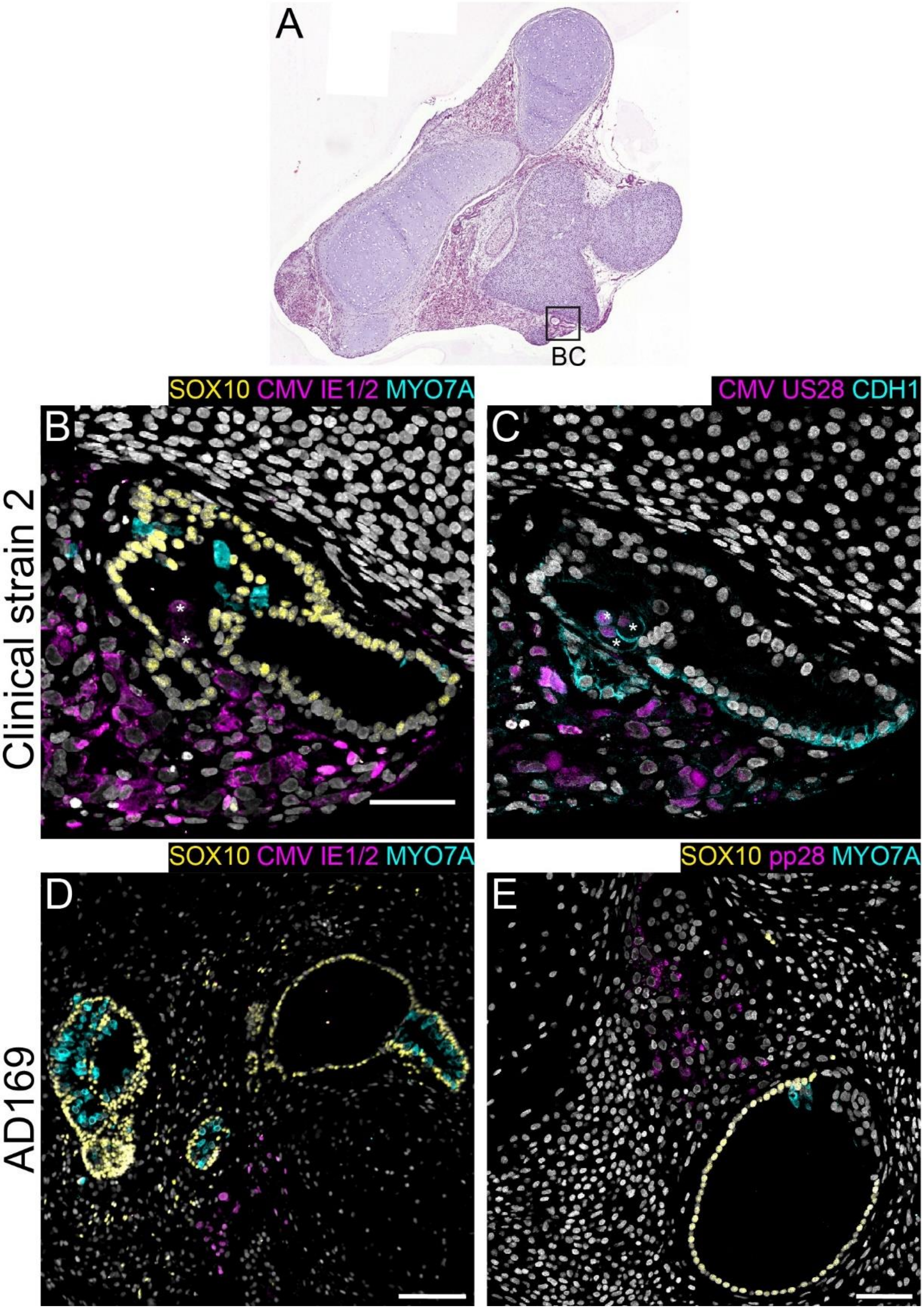

Figure S4. Clinical strain 2 shows insufficient spread with occasional infection of otic epithelial cells

Inner ear organoids were sectioned using a vibratome, inoculated with Clinical strain 2 or AD169 using an inoculum of 5.5 x 10^2 $TCID_{50}$ and cultured for 21 days.

(A) H&E staining of the whole organoid slice inoculated with Clinical strain 2. The black square indicates the otic vesicle that was immunostained in B and C, located in the periphery of the organoid.

(B) CMV IE1/IE2$^+$ (magenta) cells within the otic vesicle, indicated by asterisks, showed no expression of otic markers for hair cells (MYO7A, cyan) or nonsensory epithelial cells (SOX10, yellow) after inoculation with Clinical strain 2. Scale bar = 50 μm

(C) CMV US28$^+$ (magenta) cells within the otic vesicle, indicated by asterisks, showed co-expression with CDH1 (cyan) after inoculation with Clinical strain 2. This indicates that they are sensory or nonsensory epithelium.

(D) and (E) Vibratome sections inoculated with an AD169 dose-equivalent showing an absence of CMVIE1/IE2$^+$ (magenta) cells within the otic vesicle. D) Scale bar = 100 μm. (E) Scale bar = 50 μm.

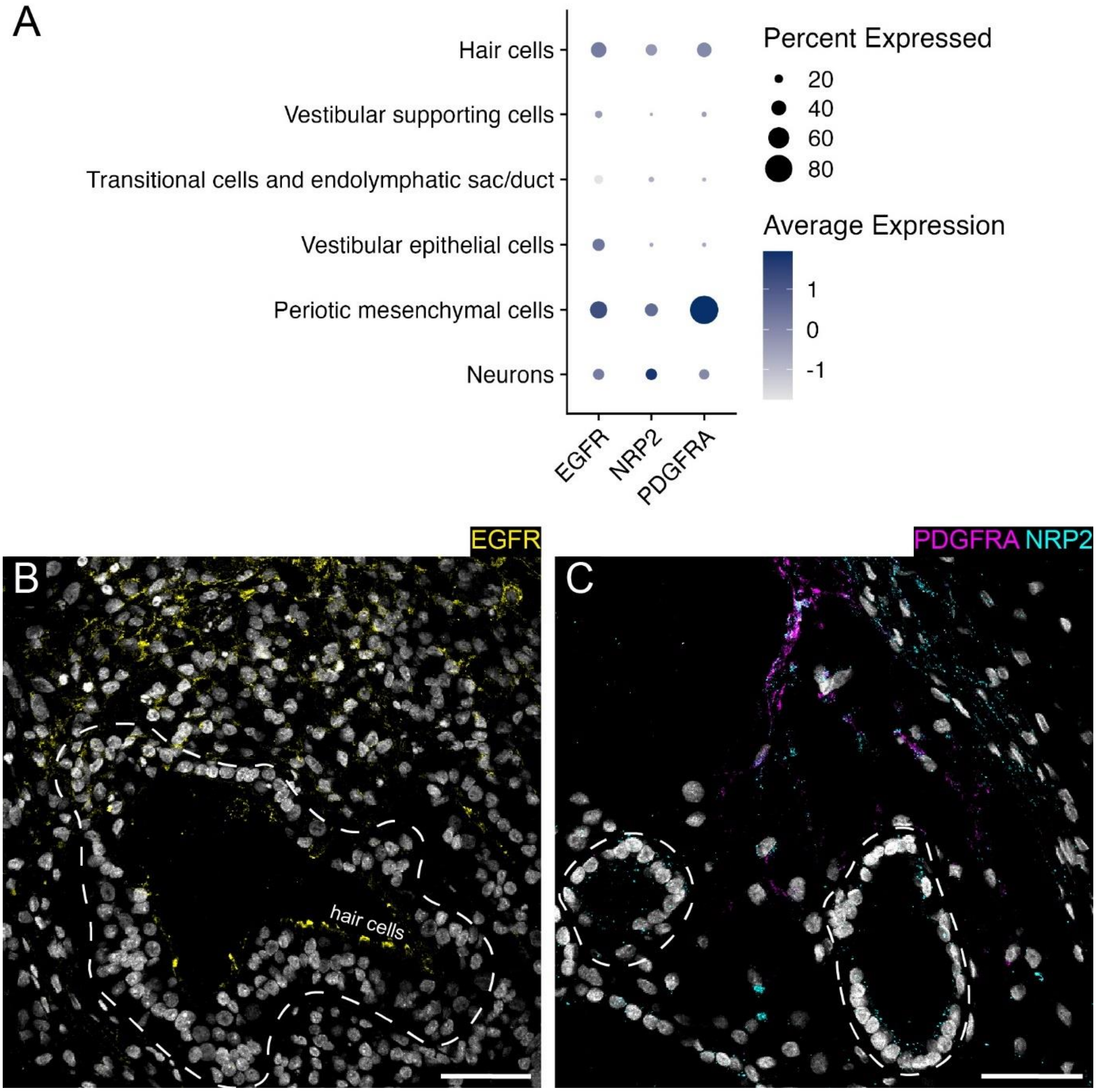

Figure S5. Cytomegalovirus entry receptors are expressed in otic cells

(A) Dot plot showing standardized gene expression of *PDGFRA* and *EGFR, NRP2* and PDGFRA in relevant inner ear cell types in the inner ear organoid

(B) Expression of EGFR protein (yellow) in the periotic mesenchyme and stereocilia. The otic vesicle epithelium is delineated by a dotted line. Scale bar = 50 µm.

(C) Punctate staining pattern of NRP2 protein (cyan) in the mesenchyme and epithelial cells. PDGFRA (magenta) expression in the mesenchymal cells. Scale bar = 50 µm.

	Organoid edge		Adjacent cells to otic vesicle		Non-sensory epithelial		Hair cells		Neurons	
	Day 7	Day 21	Day 7	Day 21	Day 7	Day 21	Day 7	Day 21	Day 7	Day 21
AD169	+++	+++	++	+++	-/+	++	-/+	+	+	+
TB40e-GFP	+++	+++	++	+++	-/+	++	-/+	++	-	+
Clinical strain 1	++	+++	++	+++	-	+	+	+	+	+
Clinical strain 2	-	+++	-	-	-	-	-	-	-	-
heat-inactivated AD169	-	-	-	-	-	-	-	-	-	-
heat-inactivated Clinical strain 2		-		-		-		-		-

Table S1. Laboratory-derived hCMV strains and clinical hCMV strains infect several inner ear cell types

Semi-quantitative assessment of the number of observed CMV-infected cells in the IEO following inoculation with different strains at 7 or 21 days post-infection. Data represent two independent experiments, comprising a total of 4–14 agarose sections per condition, each containing 3–4 vibratome slices. Scoring system: + = 1–2 CMV^+ cells; ++ = cluster of 3–10 CMV^+ cells; +++ = large cluster of >10 CMV^+ cells.
